# Neural Suppression Elicited During Motor Imagery Following the Observation of Biological Motion From Point-Light Walker Stimuli

**DOI:** 10.3389/fnhum.2021.788036

**Published:** 2022-01-07

**Authors:** Alice Grazia, Michael Wimmer, Gernot R. Müller-Putz, Selina C. Wriessnegger

**Affiliations:** ^1^Deutsches Zentrum für Neurodegenerative Erkrankungen, Rostock-Greifswald, Rostock, Germany; ^2^Department of General Psychology, University of Padova, Padua, Italy; ^3^Institute of Neural Engineering, Graz University of Technology, Graz, Austria; ^4^BioTechMed-Graz, Graz, Austria

**Keywords:** EEG, ERD/ERS, biological motion, action observation (AO), motor imagery (MI)

## Abstract

**Introduction:** Advantageous effects of biological motion (BM) detection, a low-perceptual mechanism that allows the rapid recognition and understanding of spatiotemporal characteristics of movement *via* salient kinematics information, can be amplified when combined with motor imagery (MI), i.e., the mental simulation of motor acts. According to Jeannerod’s neurostimulation theory, asynchronous firing and reduction of mu and beta rhythm oscillations, referred to as *suppression* over the sensorimotor area, are sensitive to both MI and action observation (AO) of BM. Yet, not many studies investigated the use of BM stimuli using combined AO-MI tasks. In this study, we assessed the neural response in the form of event-related synchronization and desynchronization (ERD/S) patterns following the observation of point-light-walkers and concordant MI, as compared to MI alone.

**Methods:** Twenty right-handed healthy participants accomplished the experimental task by observing BM stimuli and subsequently performing the same movement using kinesthetic MI (walking, cycling, and jumping conditions). We recorded an electroencephalogram (EEG) with 32 channels and performed time-frequency analysis on alpha (8–13 Hz) and beta (18–24 Hz) frequency bands during the MI task. A two-way repeated-measures ANOVA was performed to test statistical significance among conditions and electrodes of interest.

**Results:** The results revealed significant ERD/S patterns in the alpha frequency band between conditions and electrode positions. *Post hoc* comparisons showed significant differences between condition 1 (walking) and condition 3 (jumping) over the left primary motor cortex. For the beta band, a significantly less difference in ERD patterns (*p* < 0.01) was detected only between condition 3 (jumping) and condition 4 (reference).

**Discussion:** Our results confirmed that the observation of BM combined with MI elicits a neural suppression, although just in the case of jumping. This is in line with previous findings of AO and MI (AOMI) eliciting a neural suppression for simulated whole-body movements. In the last years, increasing evidence started to support the integration of AOMI training as an adjuvant neurorehabilitation tool in Parkinson’s disease (PD).

**Conclusion:** We concluded that using BM stimuli in AOMI training could be promising, as it promotes attention to kinematic features and imitative motor learning.

## Introduction

Motor imagery (MI), i.e., the mental rehearsal of a motor act without overt movements by muscular activity (Jeannerod, 1995, 2001), is considered to be a conscious top-down process, including the simulation of sensory, perceptual, and emotional aspects of movements (Calmels, 2019). Action observation (AO) is a bottom-up process that occurs in the presence of external stimulation (Calmels, 2019), encompassing different neurocognitive processes, such as action recognition, intention understanding, and action prediction (Sinigaglia and Rizzolatti, 2011; Vogt et al., 2013; Di Nota et al., 2017). In general, studies involving AO often employ videos of the movements of real bodies which are enriched with visual information (Mezzarobba et al., 2021). Nonetheless, the use of biological motion (BM) stimuli, i.e., point-lights that move with biologically derived kinematics (Johansson, 1973), has shown increasing benefits in AO tasks (Mezzarobba et al., 2021). First, it is an advantageous mechanism that allows the brain to recognize purposeful movements and social cues (e.g., direction, gender, and emotions) very rapidly (Beintema and Lappe, 2002; Gao et al., 2015; Lu et al., 2016; Wang et al., 2018). Second, it draws the attention of the observer to the kinematic aspects of movements and not to the unrelated elements of the action, such as the face or emotional expressions (Mezzarobba et al., 2021). Instead, the observer can focus on body postures (i.e., body sway during gait) and kinematic features (i.e., step length) without distractions (Sokolov et al., 2018; Mezzarobba et al., 2021). Third, BM triggers and spontaneously primes imitation of kinematic movements (Mather et al., 1992; Bieńkiewicz et al., 2013), hence promoting motor learning (Abbruzzese and Pelosin, 2018).

According to Jeannerod’s (2001) neurostimulation theory, AO, MI, and motor execution (ME) should be conceptualized as part of a continuum, sharing the same neural substrates (Kaneko et al., 2021). Neuroimaging data confirm this functional equivalence, by revealing the involvement of an overlapping cortical premotor-parietal network (Caspers et al., 2010; Hétu et al., 2013; Hardwick et al., 2018; Kaneko et al., 2021). This is true also for BM, which has been demonstrated to share with MI an internal simulation of the seen image, later translated into a motor representation (Ulloa and Pineda, 2007; Miller and Saygin, 2013). Neuroimaging studies revealed BM implication in a wide range of neural networks, such as the mirror neuron system (MNS) (Sinigaglia and Rizzolatti, 2011; Jeon and Lee, 2018; Thornton, 2018), suggesting an internal simulation of BM stimuli during visual perception and imagination of the actions of others (Gao et al., 2015).

Traditionally, the neural correlates of MI and AO as measured by means of electroencephalography (EEG) are the Rolandic mu rhythm, usually found within the alpha frequency band (8–13 Hz) over central electrodes positions (Pfurtscheller and Lopes Da Silva, 1999; Ulloa and Pineda, 2007; Di Nota et al., 2017; Chen et al., 2020) and the beta frequency band (18–14 Hz), associated with actual movement. The latter has been shown to desynchronize following a mental rehearsal of actions (Pfurtscheller and Lopes Da Silva, 1999; Neuper et al., 2009; Jeon et al., 2011). It is believed that, when the mu and beta rhythms desynchronize or suppress with respect to a baseline, cortical neurons over sensorimotor areas are excited (Neuper et al., 2009; Eaves et al., 2016a). Whereas, when brain rhythms synchronize, neurons are inhibited reflecting the deactivation of surrounding cortical areas that do not need to be recruited (Neuper et al., 2009; Eaves et al., 2016a). Those spatiotemporal event-related desynchronization (ERD) and event-related synchronization (ERS) usually coexist in the alpha and beta frequency bands, respectively, with more focal ERD and larger and more distributed surrounding ERS during MI, AO, and ME (Pfurtscheller et al., 2006; Wriessnegger et al., 2018).

In past studies, MI and AO tasks have been investigated separately, or either in combination (AOMI) (Eaves et al., 2016b; Di Nota et al., 2017; Scott et al., 2019). Some EEG studies revealed larger desynchronizations of the alpha and beta frequency bands over central electrodes locations during AOMI of walking as compared to the resting condition (Scott et al., 2019; Kaneko et al., 2021). Similarly, in studies analyzing the modulation of sensorimotor rhythms during the control of a brain-computer interface (BCI) for upper limbs movements, greater ERD in the lower alpha and beta bands was found during AOMI in the form of realistic neurofeedback as compared to control conditions (Neuper et al., 2009; Friesen et al., 2017). Eaves et al. also found more pronounced mu and beta suppressions over sensorimotor and parietal regions when AO and MI of static hand posture are synchronized tasks, producing stronger responses over prefrontal regions than other conditions (Eaves et al., 2016a). Consistently, EEG studies showed that BM stimuli induce the suppression of the mu wave, as compared with scrambled/non-biological movements in the areas of the MNS (Ulloa and Pineda, 2007; Gao et al., 2015). These results are in line with the hypothesis of a sensorimotor system that simulates purposeful human actions when executed, imaged, or observed (Jeannerod, 2001; Quadrelli et al., 2019).

Yet, only a few studies have been conducted using more complex imaging of whole-body movements (Wriessnegger et al., 2014). In neurofeedback research, AOMI showed a superior training effect, as reflected by an ERD amplitude enhancement (Kondo et al., 2015). We believe that the integration of AO and MI training using BM stimuli may serve as an efficient experimental paradigm to elicit increased alpha and beta neural suppression (Abbruzzese et al., 2016; Caligiore et al., 2017). Since the nature of the feedback (i.e., biological and goal-directed movements) appears to be relevant in the final neurofeedback outcome (Ulloa and Pineda, 2007; Neuper et al., 2009), the development of an effective task must include ecological stimuli that are not too enriched by irrelevant visual stimuli and that will lead to the increase in ERD during whole-body imagery, ultimately priming kinesthetic movements (Mezzarobba et al., 2021). This could benefit not only BCI systems but also more generally neurorehabilitation programs (Ono et al., 2018).

In fact, MI and AO are both considered promising tools in motor learning and neurorehabilitation, especially for stroke patients, but more recently new evidence suggests a beneficial role also for neurodegenerative diseases (Caligiore et al., 2017; Friesen et al., 2017; Dunsky and Dickstein, 2018; Calmels, 2019; Herranz-Gomez et al., 2020). Parkinson’s disease (PD), the second most common neurodegenerative disease after Alzheimer’s disease (Tolosa et al., 2021), has its main symptomatology characterized by tremors, gait impairments, and slowness of movements, the so-called *bradykinesia* (Bieńkiewicz et al., 2013). Promising evidence showed the clinical benefits of AO and MI in PD as part of an adjuvant treatment (Abbruzzese et al., 2016; LaHue et al., 2016; Caligiore et al., 2017; Miladinović et al., 2020; Lambert et al., 2021). However, since often these patients present attentional impairments, the use of BM stimuli focusing only on kinematics seems clinically advantageous (Mezzarobba et al., 2021).

This study demonstrates that MI combined with AO using BM stimuli could enhance the neural response, expressed by an increment of the desynchronization of the mu and beta frequency bands as compared to MI alone (reference condition). This study investigates which type of movement elicits a stronger neurophysiological response after combined training (AOMI). Based on the literature, we hypothesized a greater ERD of the mu and beta frequency bands (increased ERD) following BM observation and concordant MI task for three different movements (experimental conditions), as compared to when a non-BM stimulus was shown prior to the MI task (reference condition).

## Materials and Methods

### Participants

Twenty healthy right-handed volunteers (12 male, 8 female) participated in the experiment. All reported normal or corrected to normal vision, and none of them had a history of psychiatric or neurological diseases. The age of the sample ranged from 18 to 64 years old (*M* = 31; SD = ± 15). The study was approved by the local ethics committee (Medical University of Graz) and was in accordance with the ethical standards of the Declaration of Helsinki. After a detailed written and oral instruction on the paradigm and kinesthetic MI task, participants gave informed consent to participate in the experiment.

### Experimental Procedure

**FIGURE 1:**
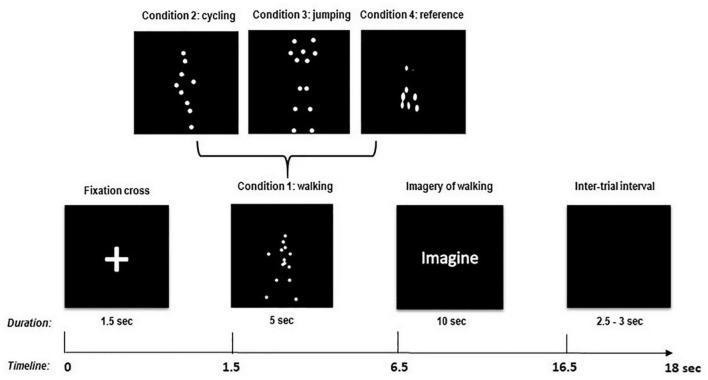
Experimental paradigm and timing of one trial.

Participants were seated in a comfortable armchair in a soundproof, air-conditioned, and dimmed room with a distance of approximately 120 cm from the 24″ full-HD monitor (60.92 cm diagonal with a resolution of 1,920 × 1,080 Pixel). The calculated visual angle of the presented stimuli was 9.5273° (stimulus height: 20 cm). They performed the task according to written instructions given prior to the experiment. Additionally, participants were verbally instructed to passively observe moving figures in the form of point-light walkers presented in a random order (BM conditions) on the computer screen. Furthermore, it was explained verbally how to perform kinesthetic imagination, making sure they could perform it before the start of the experiment. Four different conditions were presented to the participants: walking (condition “1”), cycling (condition “2”), jumping (condition “3”), and scrambled/non-biological movement (condition “4” reference). After the presentation of a fixation cross for 1.5 s, one of the 4 BM conditions appeared on the screen, followed by the written instruction “Imagine.” As long as this instruction was present on the screen (10 s), participants had to imagine with their eyes open from the first-person perspective, i.e., the physical sensations associated with the movement they just observed (kinesthetic imagery). They were also asked to keep the imagination in a repetitive and continuous manner for the entire duration of the imagery period (10 s) (Figure 1). After this imagination phase, an intertrial interval of 2.5–3 s followed before the next trial started. To make sure that the kinesthetic imagery performance was maintained over time, the experimenter required feedback from the participants between trial breaks and repeated the instructions, if needed.

Only in the scrambled movement condition (reference condition) in which no human or biological movement could be identified, participants were also asked to imagine walking (like in condition “1”). This latter instruction was given to provide a MI baseline reference for later comparisons. MI was performed 40 times per run in a pseudorandomized order. In total, four runs were performed, resulting in 160 trials (40 trials for each condition type). One trial lasted about 18 s, consisting of a fixation cross (1.5 s), the BM observation phase (5 s), the imagination phase (10 s), and an intertrial interval (2.5–3 s, randomized). Overall, each run lasted about 12 min with a 5-min break in between runs to avoid mental fatigue, for a total of 48 min to complete the whole experiment. The experimental paradigm is described in detail in Figure 1.

### Electroencephalogram Recording

The EEG signals were recorded with 32 active electrodes according to the 10/20 international system (Brain Products, Gilching, Germany). Additionally, electrooculogram (EOG) movements were recorded with three electrodes. The reference was set at electrode position FCz and the ground electrode at FPz. All signals were recorded using a BrainAmp amplifier with a sampling rate of 500 Hz.

### Preprocessing and Event-Related Desynchronization/Event-Related Synchronization Analysis

**FIGURE 2:**
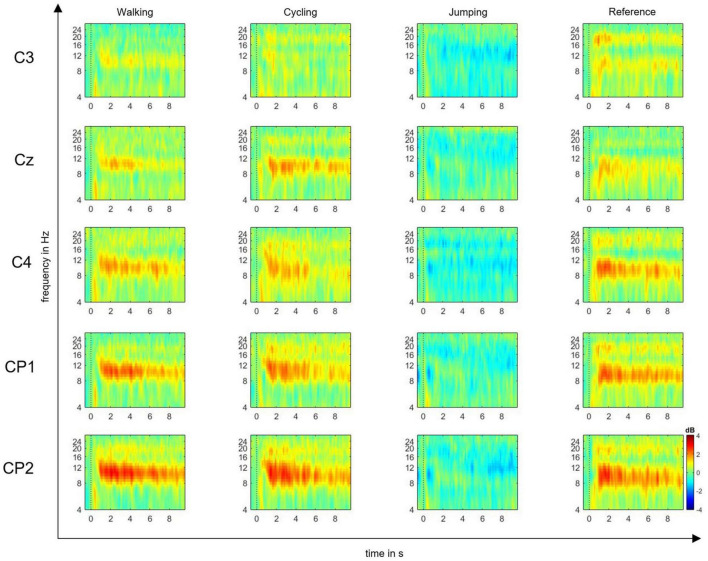
Event-related spectral perturbation (ERSP) plots. ERSP plots show mean changes in spectral power during the motor imagery (MI) epoch, relative to a –200-ms pre-stimulus baseline. Plots are shown for the electrode positions C3, Cz, C4, CP1, and CP2, and the four experimental conditions: MI of walking, cycling, jumping, and reference. The red color represents the ERS, whereas the blue color represents the ERD in decibel (dB) (range: ± 4).

The EEG signals were preprocessed using EEGLAB software (Delorme and Makeig, 2004), by first performing gross artifact detection through visual inspection. Then, a high-pass filter of 0.5 Hz and a low-pass filter of 30 Hz were applied to increase the signal-to-noise ratio. Consequently, the raw signal was downsampled from 500 to 250 Hz. The reference was maintained at FCz. For each event, the epoch length was set to −1 and 10 s within the MI time window. The baseline was selected from −200 to 0 ms prior to the imagery task. After epoching, the independent component analysis (ICA) was performed to manually exclude further artifacts from the epoched signal. Trial component rejection was conducted by both visual inspection and automatic rejection thresholds according to default parameters (Delorme and Makeig, 2004). According to the time-frequency function in EEGLAB, we computed ERD/S plots time-locked to a set of single channels: C3, CP1, CP2, Cz, C4 input epochs (10-s MI time window) (Delorme and Makeig, 2004), which are believed to mostly reflect the activity of somatosensory cortex (Pfurtscheller and Lopes Da Silva, 1999; Cho et al., 2017). EEG studies on MI and AO have in fact shown that the estimated shared cortical sources underlying the mu and beta rhythms are mainly located in central and parietal electrodes (Eaves et al., 2016a; Fox et al., 2016; Cho et al., 2017). Event-related spectral perturbation (ERSP) plots were computed to visualize mean event-related changes in spectral power (ERD/S) over time and in the selected alpha and beta frequencies for all single channels (Delorme and Makeig, 2004) (Figure 2).

We used a logarithmic scale to optimize the amplitudes of low-frequency oscillations (Herrmann et al., 2014). The significance of deviations from baseline power was computed using a bootstrap approach (values of *p* < 0.05) (Delorme and Makeig, 2004).

**FIGURE 3:**
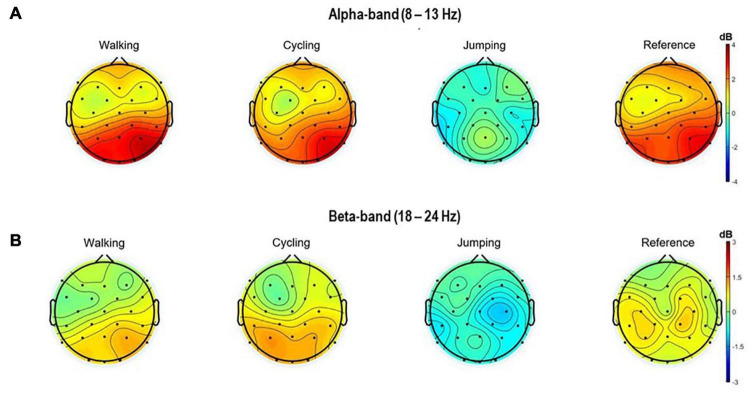
Topographical 2D scalp maps of the alpha **(A)** and beta frequency **(B)** bands for all four conditions. The maps were generated on the averaged event-related spectral perturbations of the 32 electrodes in the time window of 8.5–11 s (MI task period).

Also, the topographical 2D scalp maps of alpha and beta frequency bands (8–13 Hz and 18–24 Hz) of 3 s within the MI time window (8.5–11.5 s) were plotted (Figure 3).

### Statistical Analysis

To test the statistical significance between CONDITIONS (walking, cycling, jumping, and reference) and CHANNELS of interest (C3, CP1, CP2, Cz, C4), a two-way repeated-measures ANOVA was performed using jamovi (The jamovi project (2021) version 1.6., Retrieved from https://www.jamovi.org).

Averaged ERD/S values were analyzed for the alpha (8–13 Hz) and beta frequency bands (18–24 Hz) during a specific time window (8.5–11.5 s), considering the independent variables “CONDITION” (4 levels: walking, cycling, jumping, and reference condition) and “CHANNELS” (5 levels: C3, CP1, CP2, Cz, C4) as within-subject variables. Mauchly’s test of sphericity is used to evaluate whether the sphericity assumption has been violated. Furthermore, several *post hoc* tests with the Bonferroni correction were performed controlling the probability of making one or more Type I errors (Keselman and Rogan, 1977). A Shapiro–Wilk test was performed to test if the data are normally distributed.

## Results

The results of the Shapiro–Wilk test (α = 0.05) were non-significant, confirming a normal distribution of the dataset. Whenever sphericity was violated, the Greenhouse-Geisser correction was applied. Furthermore, the Bonferroni corrected *p*-values were used to correct for multiple comparisons with an adjusted alpha level of 0.008.

**FIGURE 4:**
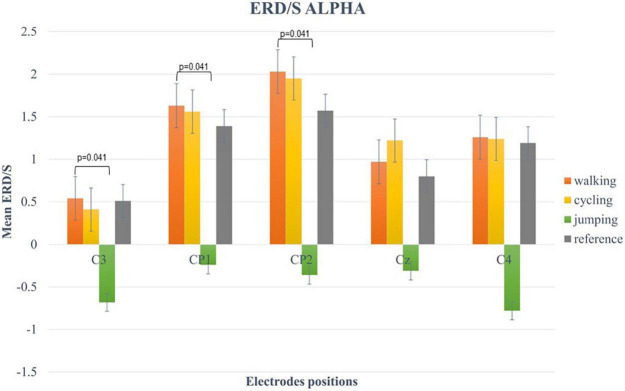
Bar plots of mean ERD/S values of the alpha-band for all conditions at electrode positions C3, CP1, CP2, Cz, C4. Significant differences were indicated by inserting the corresponding *p*-value (α < 0.05). Error bars are SE (±2).

For the alpha-band (8–13 Hz), the two-way repeated-measures ANOVA revealed a statistically significant main effect for CHANNELS (*F* (2.6, 49.3) = 7.34, *p* < 0.001, η^2^ = 0.42) and CONDITION (*F* (1.57, 29.74) = 13.63, *p* < 0.001, η^2^ = 0.28). *Post hoc* analysis showed significant differences in mean ERD/S values between Condition 1 and 3 (*t*(19) = 4.84, *p* < 0.001), Condition 2 and 3 (*t*(19) = 3.84, *p* = 0.007), and Condition 3 and 4 (*t*(19) = −3.68, *p* = 0.009) (Figure 4).

**FIGURE 5:**
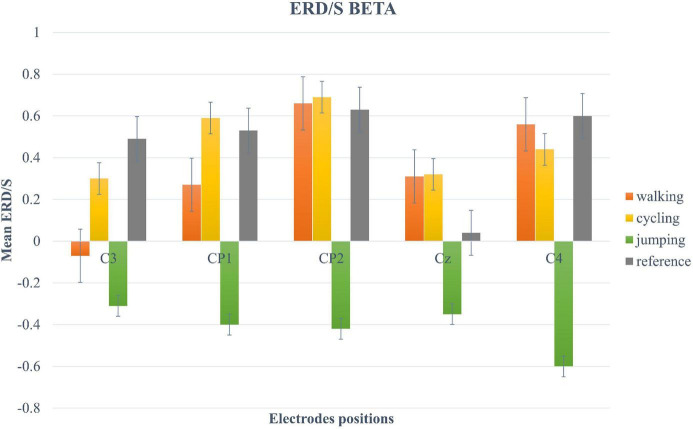
Bar plots of mean ERD/S values of the beta-band for all conditions at electrode positions C3, CP1, CP2, Cz, C4. Error bars are SE (±2).

For the beta-band, the two-way repeated-measures ANOVA revealed a significant main effect only for the within-factor CONDITION (*F* (2.12, 40.3) = 5.11, *p* = 0.009, η^2^ = 0.21). *Post hoc* tests showed a significant trend between Condition 3 and 4 (*t*(19) = −2.95, *p* = 0.049) (Figure 5).

## Discussion

### Action Observation and Motor Imagery of Biological Motion

This study defines whether AOMI using BM stimuli could produce a suppression in the mu (8–13 Hz) and beta frequency band (18–24 Hz) when compared to MI alone (reference condition). However, this seems to be true only for the observation and imagination of jumping, where an increased ERD pattern was found compared to the walking, cycling, and reference condition. It is arguable that walking and cycling are more automatic movements that can be elicited without requiring a strong involvement of cortical sensorimotor areas but rather a default mode network activation (Di Nota et al., 2017; Kaneko et al., 2021). Whereas jumping can be considered a more unusual and complex movement that needs to recruit several brain areas (Di Nota et al., 2017; Mizuguchi and Kanosue, 2017). This conjecture is supported by recent fMRI studies showing greater activation of somatosensory cortices when performing whole-body movements compared to more simple movements, such as walking (Mizuguchi and Kanosue, 2017; Carius et al., 2020). EEG studies also support this claim, by showing that compared to daily life movements, composite actions recruit a greater cognitive repertoire as reflected by greater ERDs during AO and MI of complex movements (Gonzalez-Rosa et al., 2015; Di Nota et al., 2017). These aspects are in line with the neurostimulation framework supporting the claim of an enhanced neural network activation triggered by the combination of both observation and imagery (Jeannerod, 2001; Munzert et al., 2008). In light of this, it is possible to speculate that the combination of BM and MI has an effect on the experience-dependent plasticity of alpha and beta brain oscillations by possibly modulating the shared action network patterns (Calabresi et al., 2014; Abbruzzese et al., 2016; Di Nota et al., 2017; Kaneko et al., 2021).

### Future Directions for Parkinson’s Neurorehabilitation

Neuroscientific advances suggest that the degenerated dopaminergic pathways of the basal ganglia in PD may be “by-passed” by external sensory stimulation of goal-directed actions, such as BM observation (Mezzarobba et al., 2021), producing motor improvement, the so-called “paradoxical akinesia” (Bieńkiewicz et al., 2013; Abbruzzese et al., 2016). Since parallel findings among PD and healthy participants in the ability to image kinesthetic movements of a hand after observation have been found (Bek et al., 2019), our findings suggest that training based on AOMI of complex movements could strengthen patients’ synaptic transmission, improve balance, coordination and muscle strength (Abbruzzese et al., 2016; Abbruzzese and Pelosin, 2018; Scott et al., 2021). This could be a particularly relevant training option, especially in times of pandemics, when the need for new tasks and artificial tools in telemedicine is at its highest priority (Bokolo, 2020). Even so, the clinical efficacy of AOMI training still needs to be confirmed by randomized clinical trials (Abbruzzese and Pelosin, 2018). Taken together, these results could help in defining a correct task and cognitive strategy for AOMI in neurorehabilitation based on BM (Friedrich et al., 2013; Gonzalez-Rosa et al., 2015; Caligiore et al., 2017). In fact, in this study, the imagery of jumping involved the whole-body coordination and balance, and BM stimuli were used to recruit both sensory and motor information to allow a correct coordination of upper and lower limbs at a neural level. Another interesting result, although just observational, was that a different trend for ERD distributions was found in relation to age classes. We found a more focal mu desynchronization in the youngest (18 years old) compared to a more widespread in the oldest (60 years old) participant. This is in line with another EEG study showing less lateralized activity in older participants, reflecting changes in sensorimotor functions due to aging (Zich et al., 2017b).

### Limitations

Even though the study provided promising results, there are some limitations. First, the small sample size and its age heterogeneity did not allow us to draw definitive conclusions since a certain interindividual variability or age effect could be expected when performing MI (Zich et al., 2017b; Wriessnegger et al., 2020; Ladda et al., 2021). An example of variability related to the level of expertise is the familiarity with a specific movement and the ability to produce MI (e.g., due to past sports experiences and familiarity with the MI task) (Hétu et al., 2013; Wriessnegger et al., 2014; Aoyama et al., 2020; Ladda et al., 2021). Moreover, this study did not take into account interindividual differences of ERD/S neural correlates when performing MI (Wriessnegger et al., 2020). In light of this, future studies should take into account age and interindividual characteristics, particularly when considering to provide AOMI training as a neurorehabilitation tool for patients with PD (Caligiore et al., 2017; Zich et al., 2017a). This could be carried out by including formal MI assessments at baseline (e.g., questionnaires on kinesthetic imagery) and selecting groups based on performance and age *a priori*. Ultimately, this would allow to design user-centered training protocols in line with new models of precision medicine (Friedrich et al., 2013; Hahn and Lee, 2019) and improve the application for BCIs (Friedrich et al., 2013; Turconi et al., 2014; Caligiore et al., 2017).

## Conclusion

This study provides evidence supporting the neurosimulation theory (Jeannerod, 2001; Calmels, 2019) by using abstract BM stimuli. The observation of BM of a complex act combined with MI, as in the case of jumping, induced a mu and beta suppression over the Rolandic area compared to the other types of movements and MI alone. This is believed to be in line with the existing literature on the advantage of combining AO and MI tasks (Eaves et al., 2016b). Since jumping is a kind of movement that involves the whole body, equilibrium, and coordination, it is plausible to assume that AOMI could improve both motor learning and performance (Caligiore et al., 2017). Moreover, the use of BM stimuli promotes the attentional focus on the kinematic features of movements and imitative learning (Abbruzzese et al., 2016; Mezzarobba et al., 2021), as well as brain plasticity and paradoxical akinesia phenomena in PD (Polli et al., 2017). Future research is needed to provide clinical validity for an effective visuomotor training to be included in physical rehabilitation programs or in BCI applications for patients with PD (Friedrich et al., 2013; Caligiore et al., 2017; Abbruzzese and Pelosin, 2018).

## Data Availability Statement

The raw data supporting the conclusions of this article will be made available by the authors, without undue reservation.

## Ethics Statement

The studies involving human participants were reviewed and approved by Medical University Graz. The patients/participants provided their written informed consent to participate in this study.

## Author Contributions

SW and AG: conceptualization and methodology. AG and MW: investigation and preprocessing and analysis. AG, SW, and GM-P: writing. SW: supervision. All authors had full access to all the data in the study and took responsibility for the integrity of the data and the accuracy of the data analysis.

## Conflict of Interest

The authors declare that the research was conducted in the absence of any commercial or financial relationships that could be construed as a potential conflict of interest.

## Publisher’s Note

All claims expressed in this article are solely those of the authors and do not necessarily represent those of their affiliated organizations, or those of the publisher, the editors and the reviewers. Any product that may be evaluated in this article, or claim that may be made by its manufacturer, is not guaranteed or endorsed by the publisher.
